# Evaluation of Copeptin during Pulmonary Exacerbation in Cystic Fibrosis

**DOI:** 10.1155/2019/1939740

**Published:** 2019-10-16

**Authors:** I. Wojsyk-Banaszak, P. Sobkowiak, K. Jończyk-Potoczna, B. Narożna, W. Langwiński, M. Szczepanik, Z. Kycler, A. Bręborowicz, A. Szczepankiewicz

**Affiliations:** ^1^Department of Pulmonology, Pediatric Allergy and Clinical Immunology, Poznan University of Medical Sciences, Poznań, Poland; ^2^Department of Pediatric Radiology, Poznan University of Medical Sciences, Poznań, Poland; ^3^Laboratory of Molecular and Cell Biology, Department of Pulmonology, Pediatric Allergy and Clinical Immunology, Poznan University of Medical Sciences, Poznań, Poland; ^4^Department of Pediatric Gastroenterology and Metabolic Diseases, Poznan University of Medical Sciences, Poznań, Poland

## Abstract

Copeptin was found to be a stable biomarker of inflammation and stress response in cardiac, renal, metabolic, and respiratory conditions such as pneumonia. The aim of this study was to investigate the copeptin levels in biological fluids (serum and sputum supernatant) of cystic fibrosis pediatric patients during pulmonary exacerbation and remission and to investigate the possible influence of copeptin levels on disease severity and quality of life. Copeptin serum concentrations were measured in 28 pediatric cystic fibrosis (CF) patients: 13 in stable condition and 15 during pulmonary exacerbation. In 10 CF patients, copeptin was also measured in the sputum. In all the patients, we assessed complete blood count, BMI, sputum culture, lung function, and chest imaging (with Brasfield score). The severity of symptoms was assessed using the Shwachman-Kulczycki (SK) score, and the quality of life was assessed with the Cystic Fibrosis Quality of Life Questionnaire-Revised (CFQ-R). Copeptin concentrations in serum and sputum supernatant was measured using an ELISA kit. Statistical analysis was done in Statistica v.12. Serum and sputum copeptin levels were higher in CF patients during pulmonary exacerbation than in a stable period, but the differences were not significant (*p* = 0.58 and *p* = 0.13, respectively). Copeptin did not correlate significantly with any clinical, laboratory, or spirometry markers of exacerbation. There was, however, a significant inverse correlation between the serum copeptin level and symptoms severity (*r* = ‐0.77, *p* = 0.008) and radiological changes (*r* = ‐0.5626, *p* = 0.036) during pulmonary exacerbation in pediatric CF patients. Copeptin also inversely correlated with the quality of life domains in CF patients: vitality and eating habits, mostly loss of appetite (*p* = 0.031 and *p* = 0.016, respectively). Copeptin may be useful to identify patients with a higher risk of deterioration to improve their outcomes.

## 1. Introduction

Cystic fibrosis (CF) is a multisystem congenital disease with a progressive character. It affects many organs; however, lung disease remains the major cause of patients' morbidity. Electrolyte imbalance in the bronchial lumen results in the impaired mucociliary clearance leading to chronic bacterial infection and inflammation. In the course of inflammatory process, the structure of the airways is destroyed and lung function parameters decline, both of which have a negative impact on patients' quality of life and survival [1–3]. Clinical criteria may fail to identify a proportion of patients with the deterioration of functional and structural parameters especially in its early phase, prompting efforts aimed at discovering reliable markers of disease progression [4, 5].

Copeptin (CPP) is a biomarker of prognosis and disease severity in many inflammatory, respiratory, and metabolic conditions. Copeptin is secreted in an equimolar ratio with arginine vasopressin (AVP) also known as antidiuretic hormone from the neurohypophysis, both peptides deriving from pre-provasopressin synthesized in supraoptic and paraventricular nuclei in the hypothalamus [6]. Due to its higher stability in plasma and serum, copeptin is much easier to measure than vasopressin and therefore serves as its surrogate [7, 8]. Copeptin, similar to vasopressin, participates in the regulation of the individual endogenous stress response, inflammatory response, plasma volume and osmolality, and glucose metabolism [9–11]. Increased levels of copeptin were found in adults and children with severe pneumonia [12–15] and adults with the exacerbation of chronic obstructive pulmonary disease (COPD) [16]. It was reported as a good predictor of community-acquired pneumonia (CAP) complications in children [12].

Little is known on the role of copeptin in cystic fibrosis. Given the inflammatory nature of the disease as well as multiorgan involvement including the lungs and pancreas, we hypothesized that copeptin may serve as a marker of disease exacerbation or disease progression. Moreover, we investigated if copeptin measured in a noninvasive way—from sputum—may be a predictor of pulmonary exacerbation.

## 2. Materials and Methods

### 2.1. Study Design

This was a prospective cohort study of pediatric patients with cystic fibrosis who were admitted to the Department of Pulmonology, Pediatric Allergy and Immunology, either for exacerbation or routine check-up from the 1st of September 2015 to the 31st of August 2016. The Bioethics Committee approved the study. All the parents and children participating in the study gave their written informed consent.

CF was diagnosed based on typical clinical presentation, two positive sweat chloride tests, and the presence of 2 pathogenic mutations of cystic fibrosis transmembrane regulator (*CFTR*) gene. Children born in 2007 and later were identified with newborn screening programme. The exclusion criteria were systemic glucocorticoid treatment, mycobacterial infection, autoimmune disorder, or any other severe diseases.

Exacerbation was defined, according to EuroCareCF Working Group, as the need for additional antibiotic treatment as indicated by a recent change in at least two of the following: change in sputum volume or color, increased cough, increased malaise, fatigue or lethargy, anorexia or weight loss, decrease in pulmonary function by at least 10%, radiographic changes, and increased dyspnea [17].

Copeptin serum and sputum concentrations were measured during the first 48 hours after hospital admission due to exacerbation or during control visit. Sputum samples were taken only from patients who were able to expectorate sputum, either spontaneously or after induction with hypertonic saline. In all patients, we assessed demographic parameters, inflammatory biomarkers (C-reactive protein concentration (CRP), white blood cell and neutrophil count, immunoglobulin (G), comorbidities, sputum culture, X-rays (CXR) (based on Brasfield score), computed tomography (CT) scans, pulmonary function tests results, disease severity (based on Shwachman-Kulczycki score), and quality of life (based on Specific Quality of Life Questionnaire for Cystic Fibrosis (CFQ-R)—Polish version)) [18].

### 2.2. Copeptin Measurement

Blood samples for serum were taken for tubes without anticoagulant, and after one hour, samples were centrifuged to obtain serum and frozen at -80°C for further analysis. Copeptin level in undiluted serum and sputum supernatant was measured in duplicates using an ELISA kit (Elabscience). The colorimetric analysis was performed using 450 nm wavelength using an Asys UVM340 Plate reader (Biogenet) and MicroWin software.

### 2.3. Pulmonary Function Tests

Forced expiratory volume in 1 sec (FEV_1_), forced vital capacity (FVC), and the FEV_1_/FVC ratio were measured using a Lung Test 1000 (MES, Poland) Spirometer and a Body plethysmograph according to ATS/ERS standards [19]. Residual volume (RV) and total lung capacity (TLC) were determined with the same device, in accordance with the manufacturer's guidelines.

### 2.4. Shwachman-Kulczycki and Brasfield Scores

The SK scores were calculated by two pediatric pulmonologists with expertise in CF, and CXR for both scales were evaluated by a pediatric radiologist with expertise in CF, all of whom were blinded to the copeptin results at the time of evaluation. The SK score is divided into four described domains: [1] general activity, [2] physical examination, [3] nutrition, and [4] radiological findings. Each domain is scored according to the degree of impairment. The scores of the four domains are added to obtain the final score, from which the condition of the patient is categorized as excellent (86-100), good (71-85), average (56-70), poor (41-55), or severe (<40) [20].

The Brasfield score has a maximum of 25 (normal exam) points subtracted for abnormalities in each of the following categories: air trapping, linear markings, nodular cystic lesions, large lesions, and general severity. The maximum number of points that can be subtracted in each of the categories is between 4 and 5. The minimum score in very severe form of the disease is 3 [21].

### 2.5. Cystic Fibrosis Questionnaire-Revised (CRQ-R)

CFQ-R includes nine quality of life domains, an overall health perception scale, and three symptom scales focusing on respiratory and gastrointestinal symptoms. The symptom scale describes changes in symptoms over the past 2 weeks [22]. There are four versions of the questionnaire: for young children aged 6 to 11 years, older children 12-13 years, and teenagers and adults ≥ 14 years and for parents of children younger than 14. Children and their parents answered the questions during the first 48 hours of their hospital stay.

### 2.6. Statistics

The results were expressed as the mean ± SD for numerical data with normal distribution or medians with a range for data not normally distributed. Data distribution was assessed using the Shapiro-Wilk test. To evaluate differences in copeptin levels between CF patients during exacerbation and the stable period, one-way ANOVA was used. The correlation between copeptin levels in blood and sputum and clinical parameters was analyzed using Spearman's correlation coefficient. Statistical significance was accepted at a level of 0.05. Statistical analysis was done in Statistica v.12. Power calculation was done using an online calculator freely available at https://clincalc.com/stats/Power.aspx.

## 3. Results

We measured serum copeptin concentration in 28 pediatric CF patients including 13 patients in stable condition and 15 with pulmonary exacerbation. In 10 CF patients, copeptin was also measured in the sputum supernatant. Clinical characteristics of the patients are given in Table 1.

**Figure 1:**
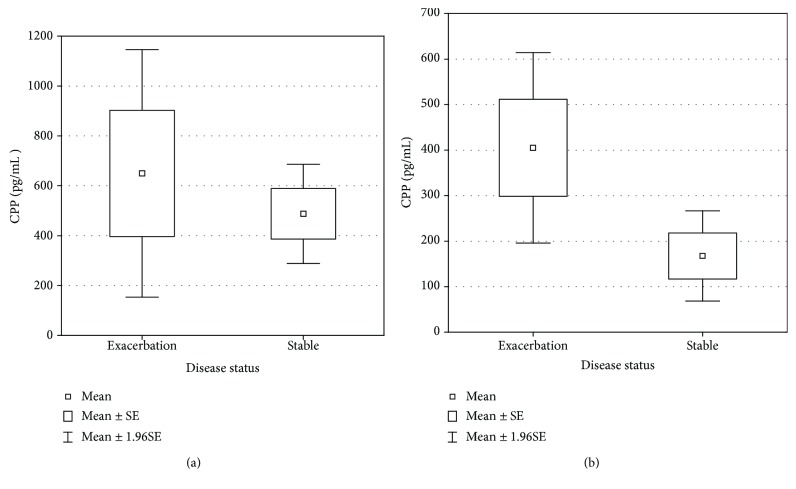
Comparison of (a) serum and (b) sputum copeptin concentration between pulmonary exacerbation and stable period (one-way analysis of variance (ANOVA)).

Serum copeptin was higher in CF patients during pulmonary exacerbation than in stable condition (649.74 ± 980.78 [pg/mL] vs. 487.76 ± 365.7 [pg/mL]; *p* = 0.58), though none of the differences reached statistical significance. Sputum copeptin was also higher in patients with pulmonary exacerbation than in stable condition (405.11 ± 261.31 [pg/mL] vs. 167.47 ± 101.20 [pg/mL]; *p* = 0.13), but the difference did not reach the statistical significance (Figure 1). The power to detect a correlation between copeptin and clinical status was 8.1% for serum and 76.7% for sputum.

**Figure 2:**
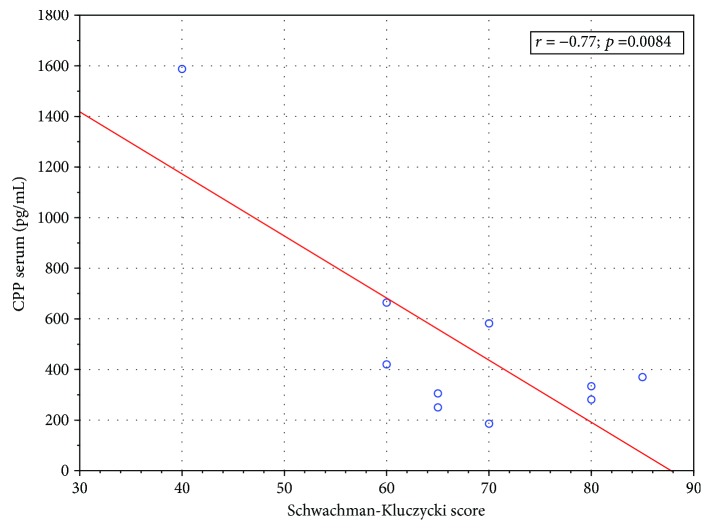
Correlation between serum copeptin level in patients with pulmonary exacerbation and Shwachman-Kulczycki score (Spearman's correlation coefficient).

**Figure 3:**
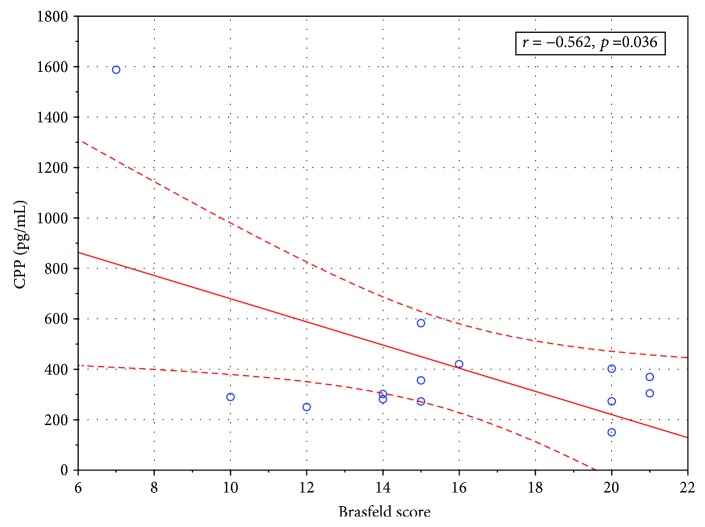
Correlation between serum copeptin level in patients with pulmonary exacerbation and Brasfield score (Spearman's correlation coefficient).

Serum copeptin did not correlate with any laboratory markers of inflammation (CRP, white blood cell count, and neutrophil count), pulmonary function tests results (FEV_1_, FVC, RV, and RV/TLC), or BMI. Serum copeptin levels were higher in girls than in boys, but only in patients during a stable period (667.80 ± 425.39 [pg/mL] vs. 266.29 ± 79.10 [pg/mL]; *p* = 0.045); the difference disappeared during pulmonary exacerbation (909.13 ± 1503.81 [pg/mL] vs. 484.43 ± 426.49 [pg/mL]; *p* = 0.43). Chronic bacterial colonization with *Pseudomonas aeruginosa* or *Staphylococcus aureus* had no influence on serum copeptin. There was, however, a significant inverse correlation between the serum copeptin level and Shwachman-Kulczycki score (*r*^2^ = ‐0.77, *p* = 0.008) (Figure 2) and Brasfield score (*r*^2^ = ‐0.5626; *p* = 0.036) (Figure 3) during exacerbation suggesting serum copeptin may correlate with the disease progression and patients' deterioration.

We also found an inverse correlation between serum copeptin and patients' quality of life reported outcomes: vitality and eating habits, mostly loss of appetite reported by parents of children younger than 14 (*r*^2^ = ‐0.47 and *p* = 0.031 and *r*^2^ = ‐0.52 and *p* = 0.016, respectively). The other domains were not significantly correlated with copeptin serum level (Table 2).

## 4. Discussion

The goal of our study was to investigate the role of copeptin in CF exacerbation as well as its potential in the disease progression monitoring. To our best knowledge, this is the first report on this biomarker in CF despite previous data on other pulmonary acute and chronic conditions in adults and children.

Cystic fibrosis is a chronic disease with inflammation occurring constantly in the airways. Copeptin was described as a marker of inflammation and stress level in many diseases [23]. Its concentrations correlate with the cortisol response to stress, but due to its higher stability, copeptin performs better [24].

The Shwachman-Kulczycki (SK) score was the first score to assess the severity of CF. It correlates with FEV_1_ results as well as chest CT scans and 6-minute walking test results [20]. Serum copeptin correlated well with disease severity estimated with SK and severity of structural lung changes reflected by the Brasfield score, the latter allowing for more detailed evaluation of both lungs on CXR [21]. More advanced disease means indeed higher stress level reflected by higher copeptin concentration.

In the studies in patients with COPD, copeptin performed well as a prognostic marker of poor prognosis in acute exacerbations as well as the stable state of the disease independently of other pulmonary risk factors [16, 25, 26]. It was superior to CRP as a predictor of COPD exacerbations [25]. Vasopressin in severe COPD has been shown to have vasoconstrictive effects induced by hypoxia [11]. Sustained hypoxemia causes downregulation of vasopressin (V1) receptors and that in turn may lead to increased vasopressin concentrations [27]. This may explain higher serum copeptin in more severely ill CF patients, even though none of them had chronic respiratory insufficiency. Short episodes of desaturation could have been experienced by those patients, especially during pulmonary exacerbations provoking copeptin release.

Du et al. [12] demonstrated that copeptin concentrations were elevated in children with CAP complicated with pleural effusion, lung abscess, pneumothorax, or pneumatocele. Plasma copeptin levels at admission are elevated in adult patients with CAP complicated by an early clinical deterioration resulting in ICU admission or death [14]. CF is characterized by a chronic bacterial infection in the airways that is exaggerated in the course of exacerbations [28]. Therefore, it seemed reasonable to expect elevated serum copeptin in CF patients during pulmonary exacerbation. Copeptin serum and sputum concentrations were indeed higher in patients with exacerbation compared to stable patients. This difference was greater in sputum than in serum thus confirming the limited extent of the infectious process and compartmentalization of inflammation [29, 30].

Scales measuring the quality of life might be useful in the evaluation of the disease progression in individual patients as they provide information about the impact of the disease on the different domains of patients' life [2]. The changes in CFQ-R respiratory mean scores were correlated with changes in spirometric indices during PE. Moreover, patients reported outcomes reflecting their perception of symptoms have an additional value consisting in describing other aspects of the disease burden than measured by physiologic variables [31]. Short-term evaluation of a chronic disease progression, especially in pediatric patients; the majority of whom having mild disease remains difficult. In such a clinical setting, seeking markers more sensitive than traditional variables including pulmonary function test results seems to be justified. Patient-related outcomes, including quality of life scales like CFQ-R questionnaire, constitute a valuable and sensitive tool to document small changes in a patient's status [31].

There was a negative correlation between parents' reported child's vitality in CFQ-R and serum copeptin in our study. Reduced vitality scores have been already reported in patients with pulmonary exacerbations [32]. We also found an inverse correlation between copeptin serum concentration and parents' reported eating disturbances. One of the frequently described clinical signs of exacerbations is the loss of appetite, though not all patients are willing to report it during medical appointments or seek medical advice should this symptom appear. These evidences support copeptin's significance as a measure of the severity of clinical disease. It may help to identify patients who would potentially benefit from more aggressive treatment.

There was no correlation between copeptin and inflammatory markers or lung function parameters. This was a bit surprising since FEV_1_, and inflammatory biomarkers such as CRP are established markers of exacerbation in CF patients [33]. Lack of correlation between copeptin concentration and lung function indices may reflect its potential in evaluating different aspects of the disease. Lung function parameters change in response to changes in airway patency due to mucus plugging or airway inflammation, while copeptin concentration may rather reflect general response to chronic stress.

Our finding concerning different serum copeptin depending on gender warrants further discussion. There was one study published on copeptin concentration in children which reported contrary results: copeptin levels have been reported to be higher in pubertal boys than pubertal girls, but the difference was nonexistent in prepubertal children [34]. The median age of our patients was 11.5 years, and considering delayed puberty in many cystic fibrosis patients, most of them were prepubertal. The difference between boys and girls in our study disappeared during PE, indicating the strong influence of inflammation on copeptin concentration, surpassing the possible differences arising from gender differences.

The complex nature of cystic fibrosis with inflammation, stress response, and decompensating multiple organs renders in difficult to find a single biomarker responsive to all the aspects of the disease. Monitoring simple inflammatory biomarkers or pulmonary function might be insufficient to account for the complex interactions leading to disease progression and patient deterioration. A biomarker of disease severity might help in stratifying patients for clinical studies. It also might be a useful tool in discussions with patients and families concerning the disease progression and outcome. As shown previously, different biomarkers might be used as complementary tools for identifying patients with a higher risk of deterioration and improve their outcomes [30]. In this context, serum and sputum copeptin levels in CF patients might provide an additional clinically relevant information.

The main limitation of our study is the small sample size in a single institutional study. The study was underpowered in regard to serum copeptin correlation; for sputum, the calculated power was close to required 80%. The strength of the study, however, is that we analyzed patients from the same institution undergoing uniform diagnostic and therapeutic regimen that guarantee a homogenous sample. Moreover, the CF patients were analyzed in different stages of the disease and we collected multiple data reflecting diverse aspects of the disease as well as the patients' perception of the disease burden.

## 5. Conclusions

We reported for the first time that copeptin concentrations correlate with CF severity measured with the Shwachman-Kulczycki score which thus may be a biomarker of symptoms severity but may not be useful to detect exacerbation. However, further studies including larger sample size are necessary to verify our findings.

## Figures and Tables

**Table 1:** Patients clinical characteristic.

Variable	Median (range) or number (%)		*p* value
	Stable	Exacerbation	
Number of subjects	13	15	—
Female	6 (46%)	7 (46%)	1.000
Age (mean)	10.4 (7-15)	13.1 (9-18)	0.018
F508 del homozygous	7 (54%)	6 (40%)	0.458
F508del heterozygous	4 (30%)	5 (33%)	0.864
BMI ≤ 3rd percentile	—	3 (13%)	0.177
BMI (median)	16.6 (14.4-21.0)	17.3 (14.03-19.5)	0.890
Pancreatic insufficiency	10 (77%)	13 (87%)	0.488
Diabetes	—	3 (0.2%)	0.087
Chronic *P. aeruginosa* infection	4 (30%)	8 (53%)	0.219
Chronic *S. aureus* infection	10 (76%)	13 (87%)	0.450
FEV1 at admission (% pred.)	100.0 (92.0-126.0)	69.5 (56.5-87.0)	<0.0001
FVC at admission (% pred.)	98.0 (89.0-111.0)	75.5 (69.5-88.0)	<0.0001
TLC (% pred.)	118.5 (112.0-124.0)	120.0 (108.5-128.5)	0.278
RV (% pred.)	187.5 (168.0-205.0)	237.5 (201.0-296.0)	<0.0001
RV/TLC (% pred.)	161.5 (136.0-176.0)	194.5 (181.5-220.0)	<0.0001
Shwachman-Kulczycki scale	85.0 (85.0-90.0)	67.5 (60.0-77.5)	<0.0001
Brasfield score	21.0 (18.0-23.0)	15.0 (13.0-20.0)	<0.001

**Table 2:** Correlation coefficients between copeptin serum level and quality of life domains (R for children older than 14 years and R-M for children under 14 years).

CFQ domain	*r* ^2^	*p*
R-physical	-0.113	0.626
R-emotion	-0.2415	0.292
R-eat	-0.0669	0.773
R-treat	-0.1934	0.401
R-social	0.1357	0.557
R-body	-0.1202	0.604
R-respir	-0.4117	0.064
R-digestive	-0.2106	0.360

R-M-physical	-0.0616	0.791
R-M-emotion	-0.34	0.132
R-M-vitality	-0.4705	0.031^∗^
R-M-school	-0.3556	0.114
R-M-eat	-0.5173	0.016^∗^
R-M-treat	-0.3348	0.138
R-M-body	-0.351	0.119
R-M-health	0.078	0.737
R-M-weight	0.2139	0.352
R-M-respir	-0.2863	0.208
R-M-digest	0.0428	0.854

∗ indicates statistical significance.

## Data Availability

The data used to support the findings of this study are available from the corresponding author upon request.
